# Myelin Impairment and Regeneration in the Central Nervous System: Molecular Mechanisms, Diseases, and Prospective Therapeutic Targets

**DOI:** 10.1002/mco2.71017

**Published:** 2026-09-20

**Authors:** Lihong Huang, Peng Cheng, Guangdong Liu, Yang Xiang, Zhen Hong, Weidong Le

**Affiliations:** ^1^ Institute of Neurology, School of Medicine, Sichuan Provincial People's Hospital University of Electronic Science and Technology of China Chengdu China; ^2^ Department of Neurology, West China Hospital Sichuan University Chengdu China; ^3^ Shanghai University of Medicine and Health Sciences Shanghai China

**Keywords:** Alzheimer's disease, biomarkers, demyelinating disorders, pathological mechanism, treatment strategies

## Abstract

Myelin plasticity is fundamental to the formation of neural networks and the optimization of neural functions, shaping circuits governing emotion, cognition, sensation, and motor control. Synthesized by oligodendrocytes, myelin undergoes lifelong remodeling that demands high metabolic activity; this renders the myelin‐oligodendrocyte unit highly susceptible to metabolic stress, aging, and pathological insults. While demyelination is the core hallmark of various chronic neurological disorders, this review specifically highlights its emerging role in Alzheimer's disease (AD). Myelin impairment is increasingly recognized as an early pathological event in AD, potentially preceding the classic accumulation of amyloid plaques and neurofibrillary tangles. Here, we provide a comprehensive overview of myelin structure and function, evaluating the potential of tracking myelin changes as early diagnostic biomarker for AD. We further synthesize recent evidence regarding myelin pathology across a spectrum of central nervous system disorders including multiple sclerosis, leukodystrophies, cerebral small vessel disease, psychiatric disorders, and traumatic injury, highlighting the underlying pathological mechanisms. Furthermore, we examine emerging therapeutic strategies, ranging from pharmacological interventions to noninvasive stimulation, aimed at restoring oligodendroglial function and promoting remyelination. Finally, we offer a forward‐looking perspective on myelin regeneration research, emphasizing its critical potential as a novel therapeutic target for AD and other neurodegenerative conditions.

## Graphical abstract

Proper myelin formation is crucial for normal neural circuit function, while myelin deficiency can lead to neural circuit dysfunction and cognitive decline, notably in Alzheimer's disease and other central nervous system demyelinating disorders. This review summarizes central myelin's structure and function, demyelination biomarkers and pathological mechanisms, and current therapeutic strategies for these disorders.

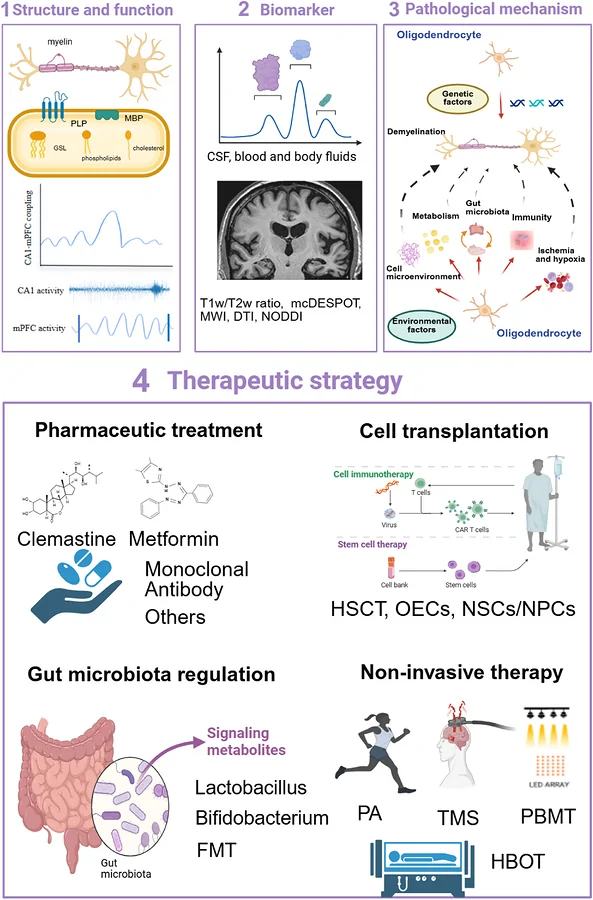

## 1 Introduction

Myelin, a multilayered lipid membrane that ensheathes neuronal axons, is produced by oligodendrocytes in the central nervous system (CNS), and is essential for rapid and efficient signal conduction. Emerging evidence indicates that myelin integrity is not only critical for nerve conduction velocity, but also deeply involved in neuronal survival, axonal metabolic support, neural circuit formation, and plasticity [1]. Consequently, damage, maldevelopment, or dysfunction of the myelin represents a central pathological feature and a key contributor to a wide range of CNS disorders [1].

With the aging of the global population, Alzheimer's disease (AD) has become a pressing public health challenge, as both its prevalence and associated economic costs continue to climb. For decades, AD research and therapeutic development have largely focused on amyloid‐β (Aβ) plaques and tau neurofibrillary tangles (NFTs) as primary hallmarkers of neuronal loss and synaptic damage [2, 3]. However, existing clinical treatments for AD exhibit limited efficacy and safety. Recent studies highlight that disruption of myelin integrity occurs early in AD and correlates closely with the rate of cognitive decline. A new model of AD now proposes myelin damage as an upstream risk factor for its hallmark pathology [4], a view supported by clinical neuroimaging and molecular studies [5, 6]. White matter hyperintensities (WMH), which reflect myelin loss, predict aggressive cognitive decline in individuals with mild cognitive impairment (MCI) [5]. Consistent with this, in vivo myelin water fraction (MWF) imaging has revealed myelin degradation in patients with MCI and dementia [7, 8], suggesting that myelin breakdown is one of the earliest structural alterations in AD brains. Notably, this process appears to be driven by age‐related DNA damage independently of amyloid deposition [6].

**FIGURE 1:**
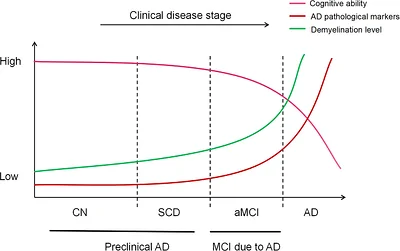
Hypothesis model diagram of AD‐related myelin injury. Preclinical AD stage includes CN and SCD. aMCI indicates MCI due to AD. AD, Alzheimer disease; CN, cognitively normal; MCI, mild cognitive impairment; SCD, subjective cognitive decline.

Critically, Depp et al. [4] proposed that age‐dependent myelin structural defect can directly or indirectly promote Aβ plaque formation via two mechanisms: one is that myelin dysfunction in the aging forebrain activates microglia, thereby impairing their capacity to clear Aβ deposits and prevent plaque formation [4]; and the other is that aging myelin loses its axon‐supportive functions, resulting in axonal distress, which subsequently elevates neuronal BACE1 and amyloid precursor protein (APP) levels, indicative of increased Aβ production [4]. Additionally, oligodendrocytes have also been shown to express high levels of APP and other amyloidogenic processing proteins [9]. When studied in App^NL‐G‐F/NL‐G‐F^ knock‐in (APPKI) mouse model of AD, oligodendrocytes have been shown to produce considerable amount of Aβ, contributing to plaque burden alongside neurons [9]. It seems that in AD, neuron is unlikely the only producer of aggregate‐forming proteins. The oligodendroglial Aβ production could explain why white matter susceptibility to the disease often occurs early [10]. Consequently, myelin breakdown and oligodendrocyte injury could be considered as a pivotal early event in AD pathogenesis (Figure 1).

Myelin's vulnerability in aging and AD stems from its dynamic biology. In the CNS, myelin is synthesized by oligodendrocytes to support axonal integrity, energy metabolism, and neural synchrony [11]. Myelin, traditionally viewed as a stable structure, is now understood to undergo dynamic modulation throughout life [12]. Its lifelong remodeling demands high metabolic activity, rendering oligodendrocytes and myelin susceptible to age‐related stressors. Also, aging myelin becomes thinner and shorter [13], increasing vulnerability to oxidative damage, Aβ toxicity, genetic and environmental risk factors [14]. This cascade culminates in axonal degeneration, white matter lesions, and circuit dysfunction underlying cognitive deficits [15]. Brain regions that contain a high myelin content has also been shown to be more resistant to developing tau pathology in AD [16], implying that myelin may confer protection against both Aβ and tau pathologies. Preclinical experiment in AD model has shown that promoting remyelination restored hippocampal sharp‐wave ripples and rescues memory [17], highlighting myelin repair as a promising therapeutic avenue for AD‐related myelin injury.

In addition of AD research highlighting the myelin's role in AD pathogenesis and its therapeutic implications, this review also synthesizes emerging evidence on various CNS diseases, including multiple sclerosis (MS), leukodystrophies, vascular dementia and cerebral small vessel disease (cSVD), psychiatric disorders, secondary myelin loss following traumatic CNS injury. We first overview the myelin biology and summarize the biomarkers of myelin, which may help detect myelin integrity. We then update the shared pathological mechanisms converging on oligodendrocyte injury and impaired myelination or maintenance, linking myelin dysfunction to several CNS diseases. Finally, we discuss therapeutic strategies aimed at promoting myelin regeneration and highlight the growing potential of myelin‐targeted interventions to slow the progression of demyelinating disorders.

## 2 Structure and Function of Myelin in Central Nervous System

The myelin has the core role from structural support to functional regulation in CNS. Thus, elucidating the fundamental structure, composition, and function of myelin is crucial for comprehending its role in AD. Furthermore, the development of sensitive and intuitive detection methods is instrumental in advancing our understanding of the role of myelin in AD.

### 2.1 Structure and Components of Myelin

Myelin in the CNS is a specialized cell membrane produced by oligodendrocytes [18]. The dry mass of CNS myelin is predominantly composed of low protein content consisting of 25%–30% [19], with a relatively high lipids content accounting for 70%–75%. Myelin contains unique lipid‐related proteins, primarily myelin basic protein (MBP) and proteolipid protein (PLP) [18]. MBP is essential for myelination [20], while PLP stabilizes myelin [21]. Myelin sheath comprises a diverse array of lipid components, primarily including cholesterol, glycosphingolipids (GSLs), and phospholipids [20]. This composition contrasts with most biological membranes, which typically exhibit a higher protein‐to‐lipid ratio. The distinctive lipid‐rich composition of the myelin confers its unique insulating properties, which are crucial for isolating axons from their surrounding microenvironment and facilitating rapid nerve conduction. Myelin is highly enriched in lipids, with a unique composition (cholesterol/phospholipids/glycolipids in a 2:2:1 ratio) called lipid rafts, which are important for the guidance of membrane proteins, trafficking, and signaling [22]. They are also central to APP amyloidosis processing in AD [23], linking altered lipid raft composition to AD pathogenesis [24]. Thus, AD can be viewed as a plasma membrane disorder [25], exploring lipid rafts can offer insights into its mechanisms and treatments.

### 2.2 Function of Myelin in Neural Oscillation and Cognition

The role of myelin in learning and memory function is important. The learning and memory functions of the brain depend on not only the information transmission between neurons, but also on the appropriate patterns or synchronization of different neuron clusters, especially the connections between the hippocampus and cortex [26, 27]. The role of myelin in learning and cognitive function can be explained from two different levels: at the cell level, myelin is responsible for insulating axons, supporting axons and ensuring bidirectional communication between axon‐glial cells. At the global brain level, myelin is responsible for coordinating the connection of neuronal components and the association of functional brain centers for complex information processing [28]. Even small changes in myelin thickness or nodal structure, could have profound effects on neuronal network function [29]. Therefore, ensuring the integrity and normal function of myelin sheaths is crucial for maintaining the accuracy and efficiency of signal transmission between neurons, as well as maintaining the normal function of neural networks.

**FIGURE 2:**
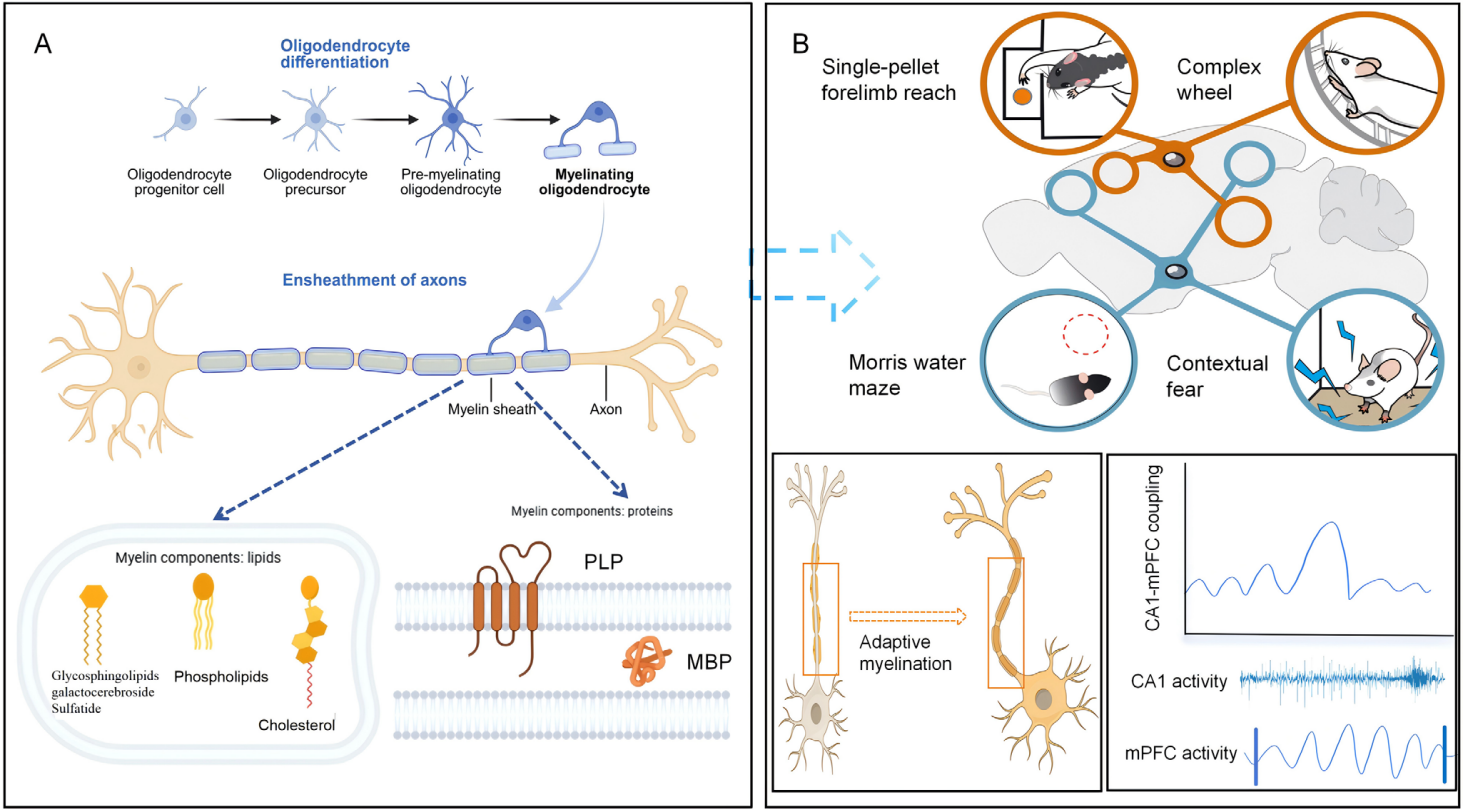
(A) represents the differentiation of OPC into oligodendrocyte to generate myelin sheath, which is mainly composed of lipids (cholesterol, GSLs and phospholipids) and proteins (PLP and MBP). (B) represents function of myelin. Stylized diagram of myelinating oligodendrocytes connecting different types of long‐term memory, including spatial memory, Pavlovian fear memory, and motor skills, assessed in specific behavioral paradigms (clockwise from top left: single‐pellet forelimb reach, complex wheel, contextual fear, Morris water maze) and underpinned by distinct neural systems [36], representing how oligodendrocyte and myelin changes in the brain are involved in the modification of circuit functions underlying learning and behavior.

The function of myelin in learning and memory was driven by experience induced oligodendrocytes generation in adulthood [30] (Figure 2). In vivo animal experiments have shown that inhibition of oligodendrocyte progenitor cell (OPC) differentiation into new myelinating oligodendrocytes can impact memory across a variety of behavioral tasks [31], while promoting adaptive myelin formation could rescue chemotherapy‐related cognitive impairment [32]. And also, the decline of learning and memory is the most important and typical clinical phenotype in various stage of AD [31]. Thus, it can be inferred that in AD, insufficient or failed adaptive myelination caused by various factors could result in learning and memory disabilities because of the synaptic and neural circuit damage [33, 34, 35]. A comprehensive understanding of the dynamics of oligodendrocyte generation and adaptive myelination is crucial for elucidating the potential role of myelin in AD [30].

### 2.3 Biomarkers of Myelin in Central Nervous System

Since this review mainly emphasized the role of demyelination in AD, we only summarize the change of the biomarkers of myelin in AD.

The role of myelin in brain function encompasses a range of biological processes that are intricately linked to the alterations in myelin lipid synthesis and composition. Consequently, the utilization of specific molecular biomarkers and imaging biomarkers to assess the integrity of the myelin sheath is valuable for the clinical evaluation of AD [37]. In this section, we evaluate the myelin‐associated molecular and imaging biomarkers in AD. The onset and progression of AD is linked to myelin damage. Biomarkers of myelin damage may serve as valuable indicators for assessing the progression of AD. Notably, reduction in myelin‐specific lipid types, such as GSL, glycerophospholipids, and cholesterol, are frequently observed in the cerebrospinal fluid (CSF) and other body fluids of AD patients [38, 39]. Also, change of lipids components was observed in various brain regions in both gray and white matter [40, 41]. These observations collectively suggest that the metabolic dysregulation of key lipid components of the myelin sheath is both stage‐specific and region‐specific in AD. Functionally, the reduction in these myelin‐specific lipids is associated with myelin degeneration and the loss of white matter integrity in the AD brain. Consequently, these specific lipid alterations may serve as biomarkers for AD‐related demyelination. Lipidomics analysis data have predicted with 90% accuracy the transition from normal cognition to amnestic MCI or mild AD within 2–3 years [42]. Moreover, MBP, PLP, and myelin oligodendrocyte glycoprotein (MOG), along with their associated antibodies, are found in serum and CSF. These components have been previously identified in certain demyelinating and neurodegenerative diseases [43]. These findings suggest the potential use of antimyelin antibodies as biomarkers for AD. As various myelin components undergo changes with aging, examining alterations in component proportions of lipid and protein profiles offers a more comprehensive approach than detection of one single component.

In comparison to the molecular biomarkers, brain magnetic resonance imaging (MRI) is regarded as a noninvasive and intuitive method for observing the microstructures of the central myelin sheath. Myelin is primarily assessed through quantitative MRI [44], which offers the measurements of white matter myelin content [45]. MRI T1 and T2 relaxation times are closely correlated with myelin content. However, the T1 signal (as observed on T1‐weighted images) is also influenced by water content and variations in white matter microstructure that are independent of myelin volume fraction [45]. Similarly, the T2 signal is affected by myelin composition or the spacing of myelin lamellae [45]. Consequently, T1w/T2w ratio images provide a proxy estimate of myelin content and are utilized as a practical approach for studying myelin content [46]. Lee et al. [47] identified significant positive correlations between the white matter T1w/T2w ratio and executive function in regions such as the fornix, sagittal stratum, anterior internal capsule, and the body of the corpus callosum. This finding suggests an association between the white matter T1w/T2w ratio and both executive function and disease progression. Recently, a novel neuroimaging technique, known as multicomponent‐driven equilibrium single pulse observation of T1 and T2 relaxation times (mcDESPOT), has been developed based on MRI T1 and T2 measurements [48]. This method offers enhanced specificity for noninvasive MRI myelin mapping. Nonetheless, mcDESPOT is also subject to certain limitations, including potential inaccuracies in assessing myelin composition and lamellar spacing, as well as the resolution of only two components [49].

In addition, it has also been reported that quantitative MRI techniques including myelin water imaging, diffusion tensor imaging (DTI), and neurite orientation dispersion and density imaging (NODDI) are often used to qualify myelin [50, 51]. Bouhrara et al. have demonstrated that individuals with MCI exhibited a reduced MWF, and such demyelination is more pronounced in AD [7]. Furthermore, a lower myelin content has been linked to a more rapid cognitive decline in a long‐term follow‐up cohort study [52], suggesting that MWF may serve as an imaging biomarker for predicting cognitive changes. DTI parameters include fractional anisotropy (FA), mean diffusivity (MD), axial diffusivity (AxD), and radial diffusivity (RD) [51]. In a recent longitudinal MRI study, Shafer et al. revealed that the rate of decline in white matter microstructure assessed by DTI, is correlated with the rate of cognitive decline [53]. Morrissey et al. also observed a reduction of FA, an indicator of white matter integrity, across the hippocampus, anterior commissure (AC), neocortex, and hypothalamus in an APPKI mouse model of AD [54]. Diffusion of DTI is regulated by a variety of tissue properties, such as axon diameter and density, myelin content, fiber orientation coherence, extracellular water, and other glial cells [55]. To compensate for this limitation, Burzynska et al. [56] employed a more sophisticated model of white matter tissue diffusion, namely, NODDI to analyze two quantitative metrics: the neurite density index (NDI) and the orientation dispersion index (ODI). Previous clinical research utilizing the NODDI model to examine cortical microstructural changes has revealed a reduction of NDI and ODI across extensive cortical regions in both young‐onset and sporadic AD participants [57, 58]. Furthermore, individuals with higher amyloid and phosphorylated tau burden exhibit lower cortical NDI [57, 58]. A recent advancement in this field is the in vivo measurement of g‐ratio defined as the ratio of the inner to the outer diameter of the myelin sheath of a myelinated axon [59]. Computing a g‐ratio metric is an effective way to interpret any combination of myelin‐weighted and axon/fiber‐weighted MR data [59]. In patients with hypomyelinating disorders, this technique could offer valuable insights into myelin sheath thickness and may be employed to monitor spatiotemporal changes as therapies become available [50].

These findings highlight the importance of myelin loss and related white matter signal assessment in cognitive function of AD patients, and can be served as an imaging biomarker to predict cognitive decline (Table 1). However, due to the low spatial resolution of standard MRI sequences [60] and the MRI methods indirectly used to assess myelin integrity [61], the understanding of the dynamics of OPC recruitment, oligodendrocyte generation, and remyelination within specific neural circuits is still limited. Therefore, more sensitive and intuitive imaging techniques and biomarkers should be further explored.

**TABLE 1:** Image biomarkers of myelin in AD and cognitive recognition.

Image biomarker	Application	Limitation
T1w/T2w ratio	T1w/T2w ratio is positively correlated with executive function in regions of the fornix, sagittal stratum, anterior internal capsule, and the corpus callosum [47].	T1 signal is influenced by water content and variations in white matter microstructure. T2 signal is affected by myelin composition or the spacing of myelin lamellae [45].
mcDESPOT	mcDESPOT enhances specificity for noninvasive MRI myelin mapping [48].	mcDESPOT has inaccuracies in assessing myelin composition and lamellar spacing, as well as the resolution of only two components [49].
Myelin water imaging	MCI and AD show decreased MWF [7]; low MWF predicts cognitive decline [52].	The whole‐brain coverage of MWF request for the high‐energy deposition and long scan times, which limited its wider dissemination [62].
DTI	Decreased FA (hippocampus, AC) indicates white matter integrity [54]; declined DTI is correlated with the rate of cognitive decline [53].	DTI is affected by axon diameter and density, myelin content, fiber orientation coherence, extracellular water, and other glial cells [55].
NODDI	Reduced NDI and ODI in cortical regions are in AD compared to CN, and lower cortical NDI is correlated with higher amyloid and phosphorylated tau burden [57, 58].	NODDI is influenced by imperfect suppression of CSF signal or by the diffusion signal arising from underlying white matter, especially in thinner areas of the cortex [58].
g‐ratio	A smaller g‐ratio indicates a thicker myelin [59]. Increased g‐ratio is exacerbated with ageing in the 5xFAD mouse model [63].	g‐ratio is influenced by MR artifacts, lack of specificity, low spatial resolution, and long acquisition times [59].

Abbreviations: AC, anterior commissure; CN, cognitively normal; CSF, cerebrospinal fluid; DTI, diffusion tensor imaging; FA, fractional anisotropy; mcDESPOT, multicomponent‐driven equilibrium single pulse observation of T1 and T2 relaxation times; MR, magnetic resonance; MWF, myelin water fraction; NDI, neurite density index; NODDI, neurite orientation dispersion and density imaging; ODI, the orientation dispersion index.

## 3 Pathological Mechanisms of Myelin Impairment in Central Nervous System Myelin‐Related Diseases

Myelin is generated by oligodendrocytes and its lifelong remodeling demands high metabolic activity, rendering oligodendrocytes and myelin susceptible to aging‐related stressors and pathological insults. Myelin impairment has been shown to be widely involved in the pathological processes of various neurological diseases, including MS, leukodystrophies, vascular dementia and cSVD, psychiatric disorders, secondary myelin loss following traumatic CNS injury, and so forth. Recently, myelin impairment is considered as an early pathological feature in AD, preceding amyloid plaque and NFT formation. Therefore, we update the evidence of myelin impairment in a range of CNS demyelinating diseases and its possible pathological mechanism, specifically in AD.

### 3.1 Pathological Mechanisms of Myelin Impairment in Alzheimer's Disease

**FIGURE 3:**
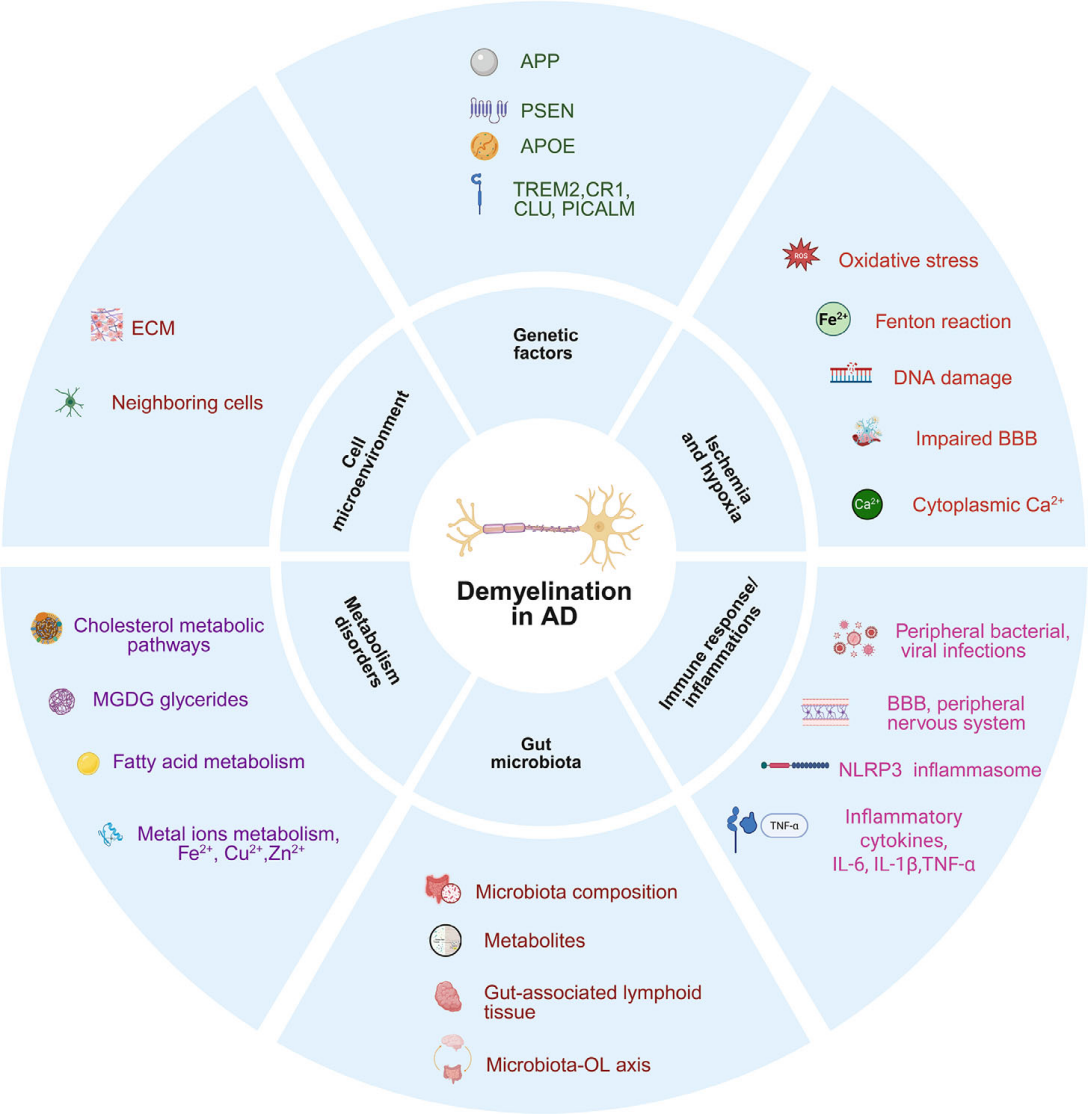
Pathological mechanism of AD demyelination. Pathological mechanism of AD demyelination included genetic factors and environmental factors. Genetic factors included APP, PSEN, APOE, and others. Environmental factors included ischemia and hypoxia, immune response/inflammation, gut microbiota, metabolic disorders, cell microenvironment are involved in the pathogenesis of AD‐related demyelination. OL, oligodendrocyte.

The pathological mechanisms of AD developed over decades, resulting in a multitheoretical framework including the Aβ hypothesis, the neuroinflammation hypothesis and the cell senescence hypothesis [64]. Among these, the amyloid cascade hypothesis stands out as a key model for understanding AD pathogenesis and guiding the development of current treatments. However, a novel working model for AD proposed that myelin damage might be as an upstream risk factor for Aβ deposition [4]. Therefore, we have summarized the pathological mechanism of myelin injury in AD for providing relevant theoretical basis for the treatment strategy of AD, especially for its early stage. Myelin loss in AD encompasses a range of alterations, such as inadequate myelin regeneration, excessive myelin degradation, and demyelination. This deterioration can be triggered by a combination of aging, genetic, and environmental factors, potentially resulting in damage of myelin or even myelin loss in AD. Aging is strongly associated with demyelination, and AD is considered as homeostatic dysfunction to age‐related myelin impairment [14]. Genetic factors contributing to this process include mutations in genes such as APP, presenilin (PSEN), Apolipoprotein (APOE), and others. Environmental factors such as ischemia and hypoxia, immunization and inflammation, alterations of gut microbiota, metabolic disorders, alterations of cell microenvironment are involved in the pathogenesis of AD‐related demyelination (see Figure 3).

#### 3.1.1 Genetic Factors

At present, genetic factors closely related to the pathogenesis of AD include mutations in genes such as APP, PSEN, APOE, and triggering receptor expressed on myeloid cells 2 (TREM2). APP gene and PSEN gene mutations are commonly found in fAD, which will directly lead to the abnormal accumulation of Aβ protein, and the formation of amyloid plaques triggers neuronal damage and myelin degeneration. Morrissey et al. found that APPKI mice display myelin microstructural changes and increased oligodendrocytes and oligodendrocyte‐related proteins, suggesting an impaired homeostasis or regulation of oligodendrocytes and myelin [54]. Minguillón Pereiro et al. found that PSEN2 mutations cause striking WMH, arguably related to myelin disruption induced by amyloid accumulation [65].

The APOE ε4 allele is recognized as one of the most significant genetic risk factors for sporadic AD. Myelin alterations in AD are more pronounced especially in individuals carrying the APOE ε4 allele [66]. The underlying mechanism may involve the association of the ApoE ε4 polymorphism with reduced cholesterol bioamyloid‐beta synthesis in myelinating oligodendrocytes [67] and accelerated demyelination with advancing age [66, 68]. Furthermore, demyelination in the white matter of AD patients can diminish the efficiency of microglia in Aβ and tau pathology [4], thereby contributing to the hallmark pathological changes and cognitive decline of AD [69, 70]. Additionally, other genes that increase the risk of AD including triggering receptor expressed on TREM2, clusterin (CLU), and phosphatidylinositol‐binding clathrin assembly protein (PICALM), all of which also affect the integrity of the myelin sheath [71, 72, 73]. However, the interaction between these genetic factors and myelin dysfunction and its contribution to AD pathology progression and cognitive decline needs further exploration.

#### 3.1.2 Ischemia and Hypoxia

Oxidative stress can be directly induced by ischemia and hypoxia, and myelinating oligodendrocytes are particularly vulnerable to oxidative stress due to their highly metabolic demands and enriched iron and lipid content [12, 74]. Consequently, ischemia and hypoxia are significant contributors to demyelination [75]. Various stages of oligodendrocytes have been reported to undergo different mechanisms of cell death [76], thereby contributing to the complexity of subcell‐type populations of oligodendrocytes and their susceptibility to diverse pathological insults. It has shown that excessive oxidative stress has a negative impact on myelin homeostasis, in decreasing OPC proliferation and maturation, oligodendrocyte numbers, and myelin‐related genes and proteins both in vitro and in vivo [77]. And its underlying mechanisms are complex. One is that cerebral ischemia and hypoxia will directly cause abnormal oxygen metabolism and affects DNA stability, induces oxidative stress, and increases iron levels [78]. Furthermore, the energy crisis caused by ischemia and hypoxia can lead to lactic acidosis, which increases the concentration of free Fe^2+^ in the cytoplasm, leading to Fenton reaction and inducing oxidative stress [79]. Moreover, oligodendrocytes exhibit the highest rate of oxidative metabolism by volume in all cell types of CNS [80] and possess a limited capacity to clear peroxides [81]. The limited compensatory ability ultimately leads to excessive production of peroxides, resulting in lipid peroxidation, loss of protein function, DNA damage, mitochondrial dysfunction, cell apoptosis, inflammatory response, and cell proliferation and differentiation defects [82, 83], ultimately leading to functional impairment of myelin [84].

Additionally, oligodendrocyte is extremely sensitive to the disruption of intracellular calcium homeostasis [85]. Under ischemic and hypoxic conditions, the resultant energy crisis and metabolic stress precipitate prolonged overstimulation of neurotransmitter receptors. This overstimulation leads to a substantial elevation in cytoplasmic Ca^2+^ levels and mitochondrial bioenergetic dysfunction [86]. Its associated characteristics include impaired oxidative phosphorylation, generation of reactive oxygen species (ROS), release of apoptotic proteins such as cytochrome C, and cell death [87, 88]. And apoptosis of oligodendrocyte directly results in injury to myelin and myelin loss. Furthermore, hypoxia can inflict damage on vascular endothelial cells, compromising the integrity of the blood–brain barrier (BBB) and exacerbating myelin damage [89]. This process is linked to the intrinsic hypoxia‐inducible factor (HIF) signaling within OPCs [90]. The interplay of these mechanisms collectively leads to myelin sheath damage under hypoxic conditions. Chronic hypoxia triggers compensatory neuroprotection via insulin‐like growth factor‐1 (IGF‐1) and HIF‐1α [91, 92]. However, severe bouts of acute hypoxia and subsequent restoration of blood flow (hypoxia/reoxygenation, H/R) overwhelm compensatory mechanisms and cause neuroaxonal damage in the brain [93] and H/R could lead to impaired myelination by upregulating HIF‐1/2 which inhibits the OPC maturation [94]. The above studies indicate that the activity of HIF signaling molecule in an appropriate oxygen environment are crucial for correct myelin formation [90]. Recently, it has been updated that ischemia and hypoxia are important factors causing abnormal oxygen metabolism in the pathogenesis of AD [95]. However, it is still unclear whether ischemia and hypoxia impair directly on myelin in AD brain, which deserves further exploration.

#### 3.1.3 Immune Response and Inflammation

The pathological framework of AD encompasses not only the traditional amyloidosis hypothesis but also the inflammation hypothesis [96]. Myelin loss was initially reported as the most important pathological feature of MS, and it has been found in several neurological diseases related to central inflammatory response [97]. In AD, this central inflammatory response is intricately linked to autoimmune processes and peripheral infections [98]. Numerous studies have indicated that specific peripheral bacteria, such as oral bacteria [99] and mycobacterium tuberculosis [100], as well as viral infections, including SARS‐CoV‐2 [101], COVID‐19 viruses [102], Epstein–Barr virus (EBV) [103], herpes simplex virus 1 (HSV‐1) [104], and herpes viruses [105], may serve as risk factors for AD.

Peripheral bacterial and viral infections exert both direct and indirect influences on the brain through various mechanisms implicated in the pathogenesis of AD [106]. Once the virus infiltrates the CNS, it begins to replicate and triggers an inflammatory response, leading to the production of inflammatory cytokines [107], such as interleukin‐6 (IL‐6), interleukin‐1β (IL‐1β), and tumor necrosis factor‐alpha (TNF‐α) [108]. These cytokines contribute to the upregulation of β‐ and γ‐secretases responsible for cleaving APP into Aβ peptides [109], and promote increased kinase activity associated with tau hyperphosphorylation [110]. Also, Aβ oligomers have potent, broad‐spectrum antimicrobial properties by forming fibrils that entrap pathogens and disrupt cell membranes [89]. Aβ fibrillization drives neuroinflammatory pathways that help fight the infection and clear β‐amyloid/pathogen deposits. In AD, chronic activation of this pathway leads to sustained inflammation and neurodegeneration [111]. It is likely that increased brain microbial burden may directly exacerbate β‐amyloid deposition, inflammation, and AD progression [111]. Furthermore, virus‐induced peripheral inflammation indirectly impacts AD pathogenesis [107]. Infections such as SARS‐CoV‐2 and COVID‐19 may exacerbate neurodegeneration in AD by enhancing neuroinflammatory, oxidative stress, and apoptosis [112]. It was recently reported that vaccination for herpes viruses reduces risk for AD dementia [105]. Thus, inflammatory and immune response play important role in the progression of AD.

There is an immune response against myelin sheaths in AD, with the emergence of autoantibodies potentially leading to damage of myelin components such as MBP [113, 114]. Furthermore, LINGO‐1 is important in myelination and LINGO 1 protein acts primarily as a negative regulator of myelination in AD [115], and intervention with the LINGO‐1 antibody has been shown to ameliorate spatial memory deficits through remyelination [115]. In the context of inflammation and demyelination in AD, inflammatory mechanisms and their products, including antibodies and macrophages, can contribute to demyelination. EBV infection has been implicated in the demyelination of the CNS [116], primarily through the activation of the NLR family pyrin domain containing protein 3 (NLRP3) inflammasome pathway [117], a critical neuroinflammatory pathway in AD [118]. Conversely, demyelination may be initiated by oligodendrocyte and myelin damage, which precedes inflammation [119], subsequently triggering local inflammatory responses such as microglial activation [120]. As oligodendrocytes express high levels of APP [9] and MAPT (tau) [121], it is possible that the accumulation of such proteins if not efficiently cleared by microglia could detrimentally affect oligodendrocytes [122, 123]. This indicates that inflammation and immune processes mediated by microglia play an important role in regulating myelin homeostasis and Aβ homeostasis in AD. Thus, there exists an intricate relationship between autoimmunity, inflammation, and demyelination in AD, and the causal processes and precise mechanisms underlying these interactions require further investigation.

#### 3.1.4 Gut Microbiota

The bidirectional regulation between the gut microbiota and the brain, commonly referred to gut–brain axis, has garnered substantial interest. A multitude of studies have established a robust correlation between gut microbiota and AD. Notable alterations in microbiota composition and diversity have been documented in 5xFAD [124], APP/PS1 [125], and APOE mouse models [126], suggesting that AD pathology may not only directly impact brain function but also exacerbate cognitive impairment through modifications in gut microbiota and associated metabolites induced by amyloid deposition. Conversely, oligodendrocyte generation and myelination may also be influenced by the gut microbiota and its metabolites [127]. Changes in myelination contributed to neurodevelopmental disorders [128]. It has demonstrated that reduced levels of hydrogen peroxide (H_2_O_2_) in milk can disrupt the gut microbiome, leading to abnormal metabolite production, including D‐glucaric acid. This metabolite has been shown to inhibit hippocampal myelin formation during infancy, potentially contributing to behavioral abnormalities observed in adulthood [129]. Furthermore, microorganisms are capable of synthesizing a diverse array of metabolites, such as short‐chain fatty acids (SCFAs), which play crucial roles in immune regulation. SCFAs have the capacity to directly influence T‐cell differentiation, promoting the development of T cells that produce proinflammatory cytokines, contingent upon the prevailing cytokine milieu, thereby eliciting an anti‐inflammatory response [130]. Additionally, SCFAs are able to traverse the BBB via transport proteins located in endothelial cells, consequently impacting neuroinflammation. This observation has led to the hypothesis [130] that self‐reactive T cells may become activated within gut‐associated lymphoid tissue as a result of molecular mimicry reactions to microbial components that resemble self‐antigens, such as MBP, PLP, and MOG, which are implicated in demyelinating diseases [131, 132]. Consequently, the microbiota–oligodendrocyte axis, particularly in relation to microbiota and MS‐associated myelin damage, has been extensively investigated [133]. However, research concerning the microbiota–oligodendrocyte axis in AD remains limited. Related research reports consistently indicate that the formation of myelin sheaths in the prefrontal cortex (PFC) occurs later in early neonatal life, making it more susceptible to gut microbiota dysbiosis [134, 135]. And the PFC region is associated with various neurological and psychiatric disorders, including cognitive impairment and autism spectrum disorder (ASD), both of which exhibit dysbiosis of the gut microbiota [136]. Therefore, treatments targeting microbiota–oligodendrocyte axis include probiotics, prebiotics, microbial metabolites, herbal bioactive compounds, and specific dietary management, could be a promising pathway for modulating oligodendrocyte homeostasis and AD‐associated demyelination [133].

#### 3.1.5 Metabolism Dysfunction

Lipids constitute a primary component of myelin, and alteration in lipid metabolism may directly cause demyelination, thereby contributing to the progression of AD [137]. A focal defect of several myelin‐associated lipid species has been identified in the hippocampus, cortex, and peripheries of the corpus callosum. This depletion is indicative of Aβ plaque‐associated disruption of the myelin sheath, attributed to the loss of myelin lipids [138]. Additionally, aberrant cholesterol metabolic pathways were observed in AD patients. Compared to controls, AD patients exhibited higher ratios of plasma 24S‐hydroxycholesterol to 27‐hydroxycholesterol, increased level of free cholesterol, and a reduced proportion of desmosterol in the brain tissue and serum [139, 140, 141, 142]. Blanchard et al. reported a disruption in lipid and cholesterol homeostasis in APOE4/4‐carrier patients and APOE4/4‐TR mice, and altered cholesterol localization in the APOE4 brain correlating with diminished myelination [67]. The expression of APOE4 in oligodendrocytes was found to promote cholesterol accumulation and impaired myelination, while treatment with cyclodextrin to enhances cholesterol transport, showed potential for the improvement of learning and executive function in aged APOE4/4‐TR mice [67]. Abnormal metabolism of monogalactosyl diglyceride (MGDG) glycerides is a significant feature of AD pathophysiology [143, 144].

It was reported that the catabolism of myelin lipids to produce ketone bodies can be utilized to meet the brain's energy and fuel requirements via pathways involving mitochondrial deficits, H_2_O_2_ production, and cPLA2 activation [145]. Furthermore, Asadollahi et al. [146] revealed that lipid turnover within the myelin sheath generates a fatty acid pool in oligodendrocytes, which may contribute to the energy balance of white matter tracts. Their findings also demonstrate that under conditions of limited glucose availability, fatty acid metabolism can support the survival of glial cells and maintain the functional integrity of myelinated axons [146]. It seems that lipid metabolism abnormality is involved in the progression of AD through various mechanisms, including promoting the aggregation and formation of Aβ, leading to abnormal cholesterol transport, and activating microglia and astrocytes, thereby triggering chronic inflammation [147]. Dynamic changes in lipid levels within specific brain regions, such as the PFC, are closely associated with cognitive deficits observed in AD. The alteration of white matter myelin‐associated lipid receptors suggests a crucial role in the pathogenesis of the disease [147]. Also, it has shown that mice with glucose metabolism disorders exhibit decreased cognitive and memory abilities, probably related to myelin damage [148]. Individuals with Type 2 diabetes (T_2_D) have a significantly higher likelihood of developing cognitive impairment, possibly due to glucose toxicity [149]. Glycotoxicity activates Cdk5, enhances cPLA2 activity and PGE2 synthesis, leading to neuroinflammation and myelin damage in the model of T_2_D mice, suggesting the potential relationship between T_2_D diabetes‐mediated demyelination and AD [149]. Therefore, abnormal glycolipid metabolism may be an important factor leading to demyelination in AD.

#### 3.1.6 Metal Ions Metabolism

Redox‐active metals such as iron, copper, and zinc, known for their capacity to generate ROS, have been identified in association with amyloid plaques and NFT, which are pathological hallmarks of AD [150, 151]. Iron, in particular, serves as a catalytic center for a variety of enzymes and is integral to numerous critical biological processes, including oxygen transport, oxidative phosphorylation, myelin production, and the synthesis and metabolism of neurotransmitters [152]. It has been reported that cortical iron levels reflect the severity of AD, with the extent of altered iron accumulation correlating with the quantity of Aβ plaques, tau pathology, and Braak stage [152]. Furthermore, Merenstein et al. employed curvature‐based analyses using quantitative susceptibility mapping (QSM) to reveal increased global magnetic susceptibility in AD compared to controls, with notable differences between the right and left hemispheres and between gyri and sulci, particularly in the temporo‐parietal regions [153]. These alterations appear to manifest subsequent to the emergence of AD pathological hallmarks, suggesting that disruptions in iron homeostasis within the brain are a significant characteristic of AD [154]. Additionally, oligodendrocytes and its myelin contain the highest iron in the brain [155], implying an inseparable relationship between iron, myelin and AD. Based on this, Bartzokis et al. [14] propose a hypothesis in AD research that the breakdown of myelin sheaths and degeneration of oligodendrocyte release a large amount of iron, further damaging to myelin and brain tissues and ultimately resulting in neurodegeneration and cognitive behavioral disorders. Therefore, precise regulation of iron homeostasis of OPC is essential for maintaining myelin and promoting remyelination.

The involvement of copper in AD has been extensively documented. In the brain tissues of both AD patients and mice models, copper deficiency has been shown to directly enhance the amyloidogenic processing of APP within cholesterol‐rich lipid rafts, thereby facilitating the formation of toxic Aβ oligomers and their aggregation into plaques in AD brain [156]. Furthermore, copper deficiency associated with demyelination indirectly accelerates the pathological progression of AD. The cuprizone model, which involves feeding mice a diet containing the toxin bis‐(cyclohexanone) oxaldehydrozone, serves as a classic model for demyelinating diseases [157]. This model induces targeted damage to oligodendrocytes, resulting in repairable demyelinating lesions across multiple brain regions [157]. Conversely, elevated copper concentration has been detected in insoluble Aβ plaques and NFT [150]. Additionally, there is an inverse relationship between total cellular copper and lipid raft copper levels, suggesting that under conditions of intracellular copper deficiency, Aβ–copper complexes are more likely to form [158]. These findings propose a novel mechanism by which cellular copper deficiency in AD may create an environment conducive to potentially harmful interactions among Aβ, copper, and cholesterol within lipid rafts, suggesting an imbalance and misdistribution of copper in the context of AD [158].

Other metal ions, including Zn [159], Se [160], and Pb [161], may affect oligodendrocyte differentiation and myelination. Zinc is one of the most abundant transition metals in the brain, second only to iron [162]. ZFP488 expression was particularly enriched in differentiated oligodendrocytes. ZFP488 can promote oligodendrocyte differentiation, and ZFP488 deficiency leads to delayed myelin formation in the developing CNS and impaired remyelination after brain demyelination injury [159]. However, their role in AD‐related demyelination remains unclear. The dysregulation of metal ions homeostasis, especially copper and iron, plays an important role in the progression of AD disease, which may be closely related to the dysfunction of oligodendrocytes and myelin. Therefore, detection of the expression of related metal ions based on metal–organic frameworks could be a promising option for the diagnosis and treatment of AD [163].

#### 3.1.7 Cell Microenvironment

Cell microenvironment can be defined as the extracellular matrix (ECM), neighboring cells, and other bioactive agents and mechanical forces that collectively influence the function of individual cells [164]. OPCs are involved in the processes of remyelination and demyelination through interactions with other cell types, such as neurons and glial cells [165, 166]. The ECM constitutes a significant portion of CNS and is integral to its normal physiological functions. ECM induces complex biological effects at various stages of myelin formation and regeneration [167]. Alterations in the ECM following AD can have profound consequences [168], and it plays a crucial role in regulating the myelin regeneration process [167]. The accumulation of Aβ in plaques may cause focal demyelination as Aβ plaques areas do not permit myelinated fibers to pass through [169]. In AD, myelin loss may be attributed to persistent toxic neuroinflammation and an inhibitory microenvironment that impedes OPC recruitment and/or differentiation [170]. The ECM and immune responses are intricately interconnected. In the context of an inflamed CNS, cytokines such as transforming growth factor‐beta (TGF‐β), TNF‐α, and interferon‐gamma (IFN‐γ) can enhance ECM synthesis, turnover, and protease secretion [171]. ECM components can function as damage‐associated molecular patterns that directly interact with specific pattern recognition receptors, such as toll‐like receptors (TLRs). Additionally, they may serve as chemotactic agents, modifying the properties and activities of immune cells, which ultimately influences myelination [167, 172]. Recent findings suggest an inhibitory role for fibulin‐2 in the experimental autoimmune encephalomyelitis model. In this model, genetic deficiency of fibulin‐2 was associated with improved behavioral recovery, facilitated by the enhanced oligodendrocyte maturation and remyelination [173]. These findings indicate that ECM component may have therapeutic potential in addressing myelin damage in AD. Future advancements are expected to yield significant insights into how ECM modifications can enhance the proliferation and differentiation of OPCs, thereby promoting myelin sheath formation and regeneration and restoring normal neural function. Furthermore, myelination is a highly regulated process involving complex interactions between oligodendrocytes and axons [174], astrocytes [175], microglia [176], respectively. This also underscores the potential of targeting the interactions between oligodendrocytes and neighboring cells as a promising strategy for repairing myelin damage in AD.

### 3.2 Pathological Mechanisms of Myelin Impairment in Other Central Nervous System Demyelinating Diseases

#### 3.2.1 Multiple Sclerosis

MS is a chronic immune‐mediated disorder of the CNS characterized by demyelination, axonal loss, and neuroinflammation, culminating in progressive neurological disability [177]. MS development requires both an appropriate genetic background and sufficient environmental triggers. Numerous genome‐wide association studies (GWASs) analysis has identified multiple genetic variations associated with the risk of developing MS [178]. And three main epigenetic modifications, including DNA methylation, histone modification, and miRNA regulation, interact and jointly participate in the pathogenesis of MS [179].

Two predominant paradigms, the “outside‐in” and “inside‐out” hypotheses, have shaped the discussion of MS origins for years [180]. The outside‐in model proposes that an aberrant autoimmune response initiated in the periphery leads to CNS damage, whereas the inside‐out model posits that a primary neurodegenerative process within the CNS triggers a secondary immune response against myelin debris. Demyelination caused by multiple pathological reasons is a key pathological process of MS.

In the development and progression of the MS, the demyelination is mainly related to the weakened regenerative ability of oligodendrocytes, which is led by its increased vulnerability to inflammatory cytokines and increased susceptibility to oxidative stress [181]. The most fundamental pathological mechanism of MS is immune‐mediated myelin damage, which is generally believed to be primarily driven by T cells and B cells [182]. The activation of CD4^+^ autoreactive T cells and their differentiation into a Th1 or Th17 phenotype are crucial events in the initial steps of MS, though many studies have shown that monocytes and monocyte‐derived macrophages are also the primary cell types responsible for cellular pathology and tissue damage. Self‐reactive T cells (Th1/Th17) are activated to attack myelin antigens (such as MBP, MOG, PLP), directly leading to myelin damage [183]. B cells produce autoantibodies (such as anti‐MOG), promoting complement activation and myelin destruction. Macrophages (M1 type) and microglia are overactivated, secreting ROS and proteases, directly damaging myelin [184]. Oxidative stress and endoplasmic reticulum (ER) stress are key factors in MS pathology. When antioxidant defense fails to neutralize excessive ROS, oxidative damage occurs, damaging lipids, proteins, and DNA within nerve cells, exacerbating mitochondrial dysfunction and maintaining chronic inflammation, accelerating disease progression. ER stress occurs when misfolded proteins overload the folding ability of organelles, triggering unfolded protein response to alleviate the crisis. Long‐term ER stress can redirect cellular signals to apoptosis, especially damaging oligodendrocytes and axons in MS [184]. And there is a bidirectional relationship between the two, where oxidative disorders disrupt ER protein folding and calcium balance, while unresolved ER stress generates more oxidative free radicals, collectively amplifying neuroinflammation, damaging glial function, and driving myelin damage [184].

#### 3.2.2 Leukodystrophies

Leukodystrophies comprise a heterogeneous group of inherited metabolic disorders caused by pathogenic mutations in genes essential for myelin homeostasis. Mutations in genes such as *ABCD1*, *PLP1*, *ARSA*, *GALC*, and *GJC2* disrupt oligodendrocyte function through diverse molecular mechanisms, ultimately leading to defective myelin formation, maintenance, or progressive degeneration of white matter [185]. Major leukodystrophy subtypes include X‐linked adrenoleukodystrophy (X‐ALD) [186], metachromatic leukodystrophy (MLD) [187], Pelizaeus–Merzbacher disease (PMD) [188], and Krabbe disease (KD) [189].

Despite marked genetic heterogeneity, emerging evidence indicates that these disorders converge on a limited number of shared cellular stress pathways. PMD, caused by mutations in *PLP1*, exemplifies disorders driven by protein misfolding [190]. Duplication of *PLP1*, the most common pathogenic variant, leads to classic PMD, whereas missense mutations frequently produce more severe phenotypes [191]. Studies using induced pluripotent stem cell‐derived oligodendrocytes from patients with PMD demonstrate that mutant PLP1 protein misfolding triggers activation of the unfolded protein response and ER stress‐mediated apoptosis [192]. Similarly, mutations in *GJC2*, which encodes connexin 47 (Cx47), cause Pelizaeus–Merzbacher‐like disease 1 [191]. Cx47 is essential for oligodendrocyte–astrocyte gap junction coupling, and its dysfunction results in ER protein retention, unfolded protein response activation, and oligodendrocyte apoptosis [193, 194]. Consistent with these findings, Cx47 knockout mice exhibit disruption of the blood–spinal cord barrier and progressive myelin loss, further highlighting the vulnerability of interglial communication networks in leukodystrophies [195].

In contrast, MLD and KD illustrate leukodystrophies driven primarily by lysosomal lipid metabolism defects. MLD results from deficiency of arylsulfatase A encoded by ARSA [196], leading to sulfatide accumulation and demyelination mediated through poly(ADP‐ribose) polymerase‐1 (PARP‐1) activation and neuroinflammatory signaling [197]. Patient‐derived iPSC models further demonstrate that sulfatide accumulation is associated with lysosomal enlargement and oxidative stress during oligodendrocyte differentiation [198]. KD, also known as globoid cell leukodystrophy, is caused by mutations in the GALC gene, which encodes the lysosomal enzyme galactosylceramidase (GALC) [199]. In *GALC*‐deficient models, transcription of myelin‐related genes remains relatively preserved; however, developmental switching of myelin‐associated glycoproteins is disrupted, and brain organoids display shortened myelin segments, indicating impaired oligodendrocyte maturation rather than complete differentiation failure [200, 201].

X‐ALD represents a leukodystrophy driven by peroxisomal metabolic dysfunction [202]. Mutations in *ABCD1*, encoding a peroxisomal ATP‐binding cassette transporter, impair the import and degradation of very‐long‐chain fatty acids (VLCFAs), which are cytotoxic to glial cells [203]. Although *ABCD1*‐mutant‐induced pluripotent stem cells can differentiate normally, mature oligodendrocytes accumulate excessive VLCFAs, particularly in severe cerebral phenotypes, suggesting stage‐specific metabolic vulnerability [204]. Furthermore, aberrant production of 25‐hydroxycholesterol in these cells activates the NLRP3 inflammasome through mitochondrial ROS and liver X receptor signaling pathways, thereby amplifying neuroinflammation [205].

Mitochondrial leukodystrophies further expand this pathogenic spectrum. Leigh syndrome, frequently caused by mutations in *NDUFS4* leading to Complex I deficiency, exemplifies disorders in which impaired oxidative phosphorylation results in ATP depletion, region‐specific gliosis, and combined neuronal and oligodendroglial degeneration [206].

#### 3.2.3 Vascular Dementia/Cerebral Small Vessel Disease

Vascular dementia is the second most common cause of dementia after AD, with cSVD representing its principal vascular substrate. Increasing evidence demonstrates that white matter lesions and myelin injury are central pathological features that critically drive the onset and progression of vascular cognitive impairment [207]. cSVD comprises a heterogeneous group of disorders with diverse etiologies, including atherosclerotic small vessel disease, cerebral amyloid angiopathy (CAA), hereditary cSVD, inflammatory and immune‐mediated cSVD, and venous collagen vascular disease [208]. Despite their etiological diversity, these conditions share common pathogenic drivers, most notably chronic hypertension, aging‐related vascular degeneration, and disease‐associated genetic mutations [208].

Hypertension represents the most significant modifiable risk factor for both cSVD and vascular dementia [209]. WMH burden correlates linearly with blood pressure levels [210], while midlife systolic blood pressure variability strongly predicts late‐life WMH progression and ventricular enlargement [211]. Sustained hypertension induces small vessel remodeling, chronic cerebral hypoperfusion, BBB disruption, and neuroinflammation, ultimately triggering caspase‐3‐mediated apoptosis of mature oligodendrocytes and subsequent myelin degeneration [212].

Aging represents the dominant and irreversible risk factor for cSVD [213], accompanied by progressive white matter degeneration and increasing WMH burden, particularly after 50 years of age [214]. Age‐related comorbidities, including hypertension, obesity, and diabetes, further accelerate white matter injury [215]. In aged experimental models, white matter integrity demonstrates heightened susceptibility to chronic hypoperfusion and hypoxia, partly due to age‐associated reductions in CREB‐dependent oligodendrogenesis [216]. Additional potentially modifiable risk factors include diabetes, obstructive sleep apnea, dyslipidemia, unhealthy lifestyle patterns, socioeconomic disparities, and COVID‐19 infection [213].

Beyond environmental and systemic risk factors, adult‐onset monogenic cSVD forms provide critical mechanistic insight into disease pathogenesis. Most hereditary cases arise from mutations in *NOTCH3*, *HTRA1*, *COL4A1*, or *COL4A2*. Among these, mutations in *NOTCH3* cause cerebral autosomal dominant arteriopathy with subcortical infarcts and leukoencephalopathy (CADASIL), the most prevalent hereditary cSVD [217, 218]. *NOTCH3* is predominantly expressed in vascular smooth muscle cells and pericytes. Pathogenic missense mutations alter cysteine residues in the extracellular domain, leading to abnormal accumulation of NOTCH3 protein and ECM components around vascular cells, likely through a toxic gain‐of‐function mechanism [219, 220].

Similarly, pathogenic variants in *HTRA1* reduce serine protease activity and cause both autosomal recessive CARASIL and more common autosomal dominant cSVD phenotypes [221]. Mutations in COL4A1 and COL4A2, which encode the α1 and α2 chains of Type IV collagen, respectively, disrupt basement membrane structure through glycine substitutions within the collagen triple‐helical domain, impairing protein folding and secretion [222]. These abnormalities result in autosomal dominant cerebrovascular disease characterized predominantly by deep intracerebral hemorrhage with marked intrafamilial phenotypic variability [223]. More recently, mutations in the 3′UTR of COL4A1 have been shown to upregulate gene expression and cause an ischemia‐predominant cSVD phenotype [224].

GWAS have identified more than 50 susceptibility loci associated with sporadic cSVD and WMH volume, highlighting molecular pathways involved in vascular development, ECM organization, and BBB regulation [225]. APOE ε4 is the strongest genetic risk factor for sporadic CAA, whereas ε2 is associated with more severe vascular pathology and increased hemorrhagic risk [226]. Individuals carrying both ε2 and ε4 alleles exhibit an additive risk profile, with the ε2/ε4 genotype representing one of the most deleterious genetic combinations in sporadic CAA [226]. Mechanistically, amyloid deposition within vessel walls disrupts BBB integrity, promotes perivascular inflammation, impairs vasoreactivity, and compromises microcirculation, leading to chronic ischemic, hypoxic, and inflammatory stress in white matter [227, 228]. These processes ultimately impair oligodendrocyte survival and function, resulting in myelin breakdown [229].

Additional susceptibility genes further reinforce the central role of BBB and oligodendroglial dysfunction in cSVD pathogenesis. Genes such as *SLC25A* and *SLC39A13*, which are highly expressed in the human BBB [230], further underscore the importance of BBB dysfunction in cSVD pathogenesis [231]. Moreover, *ULK4*, encoding a serine/threonine kinase involved in neurodevelopment, plays an essential role in oligodendrocyte maturation and myelin formation [232]. *Ulk4* deficiency mice demonstrate reduced expression of oligodendrocyte lineage transcription factors and approximately 50% reductions in myelin production, further linking genetic susceptibility to white matter degeneration [232].

#### 3.2.4 Psychiatric Disorders

Increasing evidence indicates that myelin dysfunction represents a shared pathological substrate across multiple psychiatric disorders, including anxiety disorders, major depressive disorder (MDD), schizophrenia (SCZ), and ASD [233]. These conditions arise from complex interactions among genetic susceptibility, environmental stressors, immune dysregulation, microbiome‐derived signals, and metabolic disturbances, which converge on oligodendrocyte lineage dysfunction and impaired myelin homeostasis.

GWAS studies have identified numerous psychiatric susceptibility genes that directly regulate oligodendrocyte development and myelination [234, 235]. The SCZ risk gene *DISC1*, a downstream effector of ERBB2/ERBB3 signaling, exhibits increased expression of splice variants, including *DISC1‐Δ3*, *DISC1‐Δ7Δ8*, and *DISC1* Esv1, in affected individuals [236]. OPC‐specific overexpression of DISC1‐Δ3 in transgenic mice induces cellular hypertrophy, increased branching complexity, synaptic deficits, and SCZ‐like behaviors [237]. Another SCZ‐associated gene, *TMEM108*, suppresses OPC proliferation and delays myelination, whereas gene deletion results in corpus callosum hypermyelination [238]. In ASD, haploinsufficiency of the chromatin remodeler *CHD8* represents one of the strongest genetic risk factors [239, 240, 241]. *CHD8* deficiency directly impairs OPC differentiation and induces social and repetitive behavioral abnormalities in mice [242]. Similarly, loss of the ASD‐associated synaptic scaffold gene *SHANK3* produces enhanced OPC proliferation but impaired oligodendrocyte maturation and functional myelination [243]. Collectively, these findings support the concept that psychiatric risk genes frequently converge on oligodendroglial lineage regulation and myelin integrity.

Environmental and developmental stressors further amplify oligodendrocyte vulnerability. Early‐life adversity, including parental deprivation, disrupts myelin development and impairs PFC‐dependent cognitive function [244]. Prenatal insults such as infection, malnutrition, and maternal stress activate maternal immune signaling and alter placental endocrine regulation, thereby interfering with fetal oligodendrocyte development and increasing psychiatric susceptibility in offspring [245, 246]. Chronic psychosocial stress and childhood trauma disrupt hypothalamic–pituitary–adrenal axis signaling and cortisol regulation [247], resulting in oligodendrocyte structural abnormalities, neural circuit dysfunction, and affective behavioral disturbances in rodent models, with parallels observed in human psychiatric conditions [248, 249, 250]. Epigenetic regulation represents a key mechanistic interface linking environmental exposure to myelin dysfunction. In social isolation models, reduced levels of the repressive histone modification H3K9me3 in prefrontal oligodendrocytes lead to dysregulated myelin gene expression, whereas the atypical antipsychotic quetiapine restores H3K9me3 levels, promotes remyelination, and improves behavioral abnormalities [251].

Neuroimmune activation constitutes a central pathogenic hub integrating genetic and environmental risk factors. Neuroinflammation is increasingly recognized as a shared pathological feature across MDD, anxiety disorders, ASD, and SCZ [252, 253]. Microglial activation with rapid phenotypic and functional reprogramming is frequently observed [254] accompanied by astrocytic reactivity [255]. In SCZ, approximately 70% of patients exhibit reactive astrogliosis with heterogeneous elevation of astrocytic markers [256]. In ASD, total astrocyte numbers are reduced, yet the proportion of activated astrocytes is increased [257]. Accumulating evidence further indicates that oligodendrocyte lineage cells actively participate in neuroimmune signaling rather than functioning as passive targets [258]. Oligodendrocyte lineage cells can function as antigen‐presenting cells and produce MHC I/II molecules, cytokines, chemokines, and complement regulators during CNS inflammation [259]. Oligodendrocytes and OPCs can express MHC Class I and II molecules and produce cytokines, chemokines, and complement regulators during inflammatory responses [259, 260]. OPCs recruit T cells through secretion of CCL2, CCL5, and CXCL10 and modulate T‐cell responses through MHC expression, PD‐L1‐mediated inhibition, or Fas ligand‐induced apoptosis [261, 262]. Expression of TNFR1 and TNFR2 on OPCs further suggests context‐dependent pro‐ and anti‐inflammatory functions [263]. Single‐cell transcriptomic analyses have identified a population of immune‐activated oligodendrocytes in MDD characterized by immune gene expression and impaired myelin regulatory programs [264]. These cells are also observed in chronic social defeat stress models and express markers including MHC II, C3, and P2RY12 directly linking oligodendrocyte immune phenotypes to psychiatric pathology [264].

The gut microbiota is a critical regulator of myelin homeostasis, coordinating metabolic signaling, immune responses, and neuroinflammation [265]. Accordingly, the gut–brain axis provides a mechanistic framework for understanding psychiatric disorders [266, 267]. SCFAs, produced by microbial fermentation of dietary fiber, are critical regulators of myelin formation. Altered SCFA levels are increasingly recognized as a mechanistic link connecting gut dysbiosis to CNS myelin dysfunction in psychiatric disorders [268]. The PFC, a key region in psychiatric pathology, undergoes extended myelination into early adulthood (25–30 years). This prolonged developmental window makes the PFC especially susceptible to microbiome‐mediated metabolic and immune modulation [269]. Microbiome dysregulation contributes to white matter demyelination via toxic metabolites [270]. Dysbiosis‐related endotoxins further induce systemic inflammation, activate central glial cells, compromise BBB integrity, and culminate in white matter demyelination [271].

#### 3.2.5 Traumatic Brain Injury

Traumatic brain injury (TBI) and spinal cord injury (SCI) initiate overlapping secondary pathological cascades following primary mechanical damage, including oxidative stress, excitotoxicity, neuroinflammation, and metabolic dysfunction [272, 273]. These processes progress over days to weeks, resulting in widespread oligodendrocyte loss, demyelination, impaired OPC differentiation, and remyelination failure [274]. Mature oligodendrocytes are particularly susceptible to oxidative stress, excitotoxicity, ER stress, and mitochondrial dysfunction. Sequential or synergistic activation of these mechanisms compromises oligodendrocyte function and triggers cell death. Oxidative stress, mediated by reactive oxygen and nitrogen species, damages DNA, proteins, and lipids, rendering both newly generated and mature oligodendrocytes prone to apoptosis [275]. Excitotoxicity via excessive glutamate activation of AMPA/kainate receptors induces intracellular calcium overload, further activating caspase‐dependent apoptotic pathways [276]. ER stress and mitochondrial dysfunction contribute additionally, as calcium release from the ER and mitochondrial accumulation reduce membrane potential, trigger cytochrome c release, and activate downstream apoptosis [276, 277].

CNS trauma rapidly activates microglia and astrocytes and facilitates infiltration of peripheral immune cells, which release proinflammatory cytokines, ROS, and proteases that directly damage myelin [278, 279, 280]. Following SCI, proinflammatory microglia are rapidly activated and exert a dual role in remyelination: initially, they phagocytose myelin debris, creating a favorable environment for OPC recruitment and differentiation [281], and secrete TNF‐α, stimulating the generation of premyelinating oligodendrocytes [282]. However, excessive or sustained proinflammatory signaling is cytotoxic to mature oligodendrocytes, and OPCs are particularly vulnerable, ultimately impairing remyelination [283, 284, 285]. A complex network forms among microglia, astrocytes, and oligodendrocytes postinjury, collectively regulating myelin preservation and damage, thereby influencing repair outcomes [286, 287].

Oligodendrocyte loss after CNS injury precipitates demyelination. Although OPCs exhibit rapid and sustained proliferation postinjury, especially at lesion borders, and migrate toward sites of damage, their reparative potential is often constrained by the glial scar [288, 289]. This scar, composed of reactive astrocytes, activated microglia, and ECM enriched with inhibitory molecules, creates a microenvironment that impedes regeneration [290]. OPC differentiation into mature oligodendrocytes is suppressed within this environment through multiple mechanisms, including aberrant Wnt pathway activation, myelin regeneration inhibitors (e.g., Nogo‐A, LINGO‐1), and deposition of inhibitory ECM components [291, 292].

## 4 Treatment Strategies for Myelin Impairment

### 4.1 Treatment Strategies for Myelin Impairment in Alzheimer's Disease

At present, the treatment and prevention strategies of myelin sheath injury are generally focused on promoting the oligodendroglial functions and myelination, which include pharmaceutics treatments and noninvasive physical or brain stimulation therapies. Pharmaceutic treatment included Clemastine, Metformin, Donepezil, drugs on Nogo‐A and LINGO‐1 signal pathway, drugs on nuclear receptor retinoid X receptor (RXR) signaling pathway, drugs on GABAergic and glutamine signaling pathway. Additionally, the noninvasive therapies for AD‐related demyelination, such as physical activity (PA) and transcranial magnetic stimulation (TMS), photobiomodulation therapy (PBMT) and hyperbaric oxygen therapy (HBOT) suggest the potential for broader applications [293].

#### 4.1.1 Clemastine

Clemastine, a pharmacological agent known to enhance myelin formation, has demonstrated therapeutic potential in the treatment of various neurological disorders [294, 295, 296]. Its mechanism may be mediated through the M1 muscarinic receptor on OPCs [297], and may also directly regulate Olig2 expression, thereby facilitating OPC differentiation and myelination [298]. Furthermore, it may be achieved by regulating the mitochondrial associated fas–casp8–bid axis to increase the proportion of myelinated oligodendrocytes in OPC, thereby inhibiting apoptosis of premyelinated oligodendrocytes [299]. Recent research has demonstrated that clemastine can enhance myelination and improve cognitive function in AD mouse models, and there are several clinical trials currently under investigation [300]. While it has shown promise as a remyelinating agent in MS clinical trials, its application for white matter demyelination in AD requires further investigation.

#### 4.1.2 Metformin

It has been well‐established that the dysfunction of insulin and IGF signaling network in brains is a mediator of dysregulated energy metabolism, and is probably a major driver of neurodegenerative cascade in AD [301]. Recent studies have demonstrated that the diabetes medication metformin, a potent activator of the cellular energy sensor AMPK complex, facilitates recovery from demyelination. This is achieved by its pleiotropic effects including the changes of metabolic reprogramming of oligodendrocytes, antioxidant and anti‐inflammatory effects, enhancement of oligodendrogenesis, promotion of neural stem cells (NSCs) survival and proliferation, and myelination augmentation [302, 303, 304]. Xie et al. [305] reported that metformin increased the expression of MBP, Olig2 and CC1, enhanced the thickness and density of the myelin sheath, and reduced oxidative stress and microglial infiltration following chronic hypoxia induction. These effects may play a role in the improvement of learning and memory, and motor abilities in mice with chronic hypoxia‐induced white matter injury [305].

#### 4.1.3 Donepezil

Donepezil, an acetylcholinesterase inhibitor approved by the Food and Drug Administration (FDA) for the treatment of AD, has also been investigated for its potential myelin‐promoting properties [306, 307]. Both in vitro and in vivo studies have shown that donepezil facilitates the differentiation of OPCs into oligodendrocytes, enhances myelin formation, and upregulates myelin‐specific proteins [308]. The PI3K/Akt/mTOR signaling pathway is implicated as a significant contributor to its regulatory mechanism. Furthermore, Imamura et al. have demonstrated that donepezil‐induced oligodendrogenesis is mediated through both estrogen receptor subtypes, ERα and ERβ [309]. These findings suggest that donepezil promotes myelin formation through multiple mechanisms; however, the precise mechanisms in AD remain unclear, requiring further investigation.

#### 4.1.4 Drugs on Nogo‐A and LINGO‐1 Signal Pathway

Two distinct negative regulators of myelination, Nogo‐A and LINGO‐1 proteins, have been identified as promising targets for promoting remyelination and regeneration. Consequently, antagonists of Nogo‐A or LINGO‐1 have been proposed as potential therapeutic strategies for the treatment of demyelinating diseases [292]. Dl‐3‐butylphthalide (dl‐NBP) has demonstrated the ability to enhance OPC proliferation through the Nogo‐A [310] and promote remyelination via modulation of AMPK/SIRT1 and STAT3/NF‐κB signaling [311], and it has been shown to alleviate neuropathology associated with AD [312]. Furthermore, anti‐LINGO‐1 therapy has been reported to improve spatial learning and partially restore MBP levels, while genetic deletion of LINGO‐1 in animal models has been found enhancing remyelination [313].

#### 4.1.5 Drugs on Nuclear Receptor Retinoid X Receptor Signaling Pathway

The RXR signaling pathway enhances the expression of ATP‐binding cassette transporter A1 (ABCA1) and APOE, thereby directly facilitating the maturation of oligodendrocytes, which in turn accelerates myelin regeneration and ameliorates cognitive functions associated with AD [314, 315]. Furthermore, activation of RXR can stimulate monocytes, macrophages, and microglia to clear myelin debris, thereby promoting myelin regeneration [316]. Bexarotene, a synthetic RXR agonist, has been shown to increase the expression of oligodendrocyte markers such as Olig2, O4, and MBP in specific regions of the hippocampus and cortex. This suggests that RXR agonists‐induced remyelination through the proliferation and maturation of OPCs, may contribute to the observed cognitive improvements in 3xTg‐AD mice [314].

#### 4.1.6 Drugs on GABAergic and Glutamine Signaling Pathway

GABAergic and glutamine signaling pathways play critical roles in regulating oligodendrocyte development and myelin integrity, offering potential therapeutic targets for myelin repair in AD. GABA signaling, particularly via the GABA(B) receptor, promotes OPC differentiation and myelination, as evidenced by the promyelinating effects of the agonist baclofen [317]. In AD, Aβ and tau toxicity disrupt glutamine signaling, leading to impaired glutamate clearance, excitotoxic damage to oligodendrocytes, and myelin injury [318, 319]. Restoring glial glutamate transporter function has been shown to ameliorate these deficits [320]. These findings highlight that modulating GABAergic and glutamine pathways represents a promising strategy to promote oligodendrocyte function and myelin regeneration in AD.

The pharmaceutic treatment strategies for myelin damage in AD are summarized (see Table 2), emphasizing the promotion of myelin regeneration through various mechanisms such as enhancing the differentiation of OPCs, maturation of oligodendrocytes, and myelin formation to repair white matter damage, which is expected to alleviate the AD. Overall, these findings provide a scientific basis for myelin‐targeted therapies for AD, but further experiments and clinical trials are needed to validate its efficacy and safety.

**TABLE 2:** Pharmaceutic treatment for myelin damage in AD and other cognitive impairment disease.

Drug	Target pathway and mechanism	Key effects	Experimental model
Clemastine	Antagonizes Chrm1 receptor on OPCs → Olig2↑ → OPC differentiation↑ → myelin renewal↑	Occurrence and peak frequency of hippocampal sharp wave ripples↑, cognitive function↑	APP/PS1 mice model [17]
Metformin	Activation of NRF2/HO‐1/NF‐κB → inhibiting oxidative stress and inflammation → MBP↑, Olig2↑, CC1↑, PDGFRα↑, NG2↑, MAG↑, and CNPase↑ → Myelin density↑, differentiation, and maturation of oligodendrocytes↑	Short‐term learning and memory↑	WMI mice [305]
Dl‐NBP	AMPK/SIRT1↑ and STAT3/NF‐κB↓ → Olig2↑, MBP↑ → OPC differentiation↑ and myelin integrity↑	Spatial learning and memory performance↑	CCH mice [311]
LINGO‐1 antagonist	LINGO‐1 expression↓ → CNPase^+^ cells↑, Olig2^+^/PDGFR‐α^+^ cells↓, binary area of MBP↑ → OPC proliferation and maturation↑ and myelination↑	Learning and memory abilities↑	12‐month‐old APP/PS1 mice [321]
Bexarotene	Activation of RXR↑ → Olig2^+^ cells and area↑, O4^+^cells and area↑, MBP^+^ cells and area↑ → OPC proliferation and maturation↑ and myelination↑	Cognitive function↑	24‐month‐old 3xTg‐AD mice [314]
GABAergic signaling pathway	Activation of GABA(B) receptor → CNPase↑, g‐ratio↑, PDGFR‐α^+^ cells↑ and CC1^+^ cells↑ → OPC proliferation and differentiation↑	Long‐term memory↑	Sevoflurane ‐induced impairment of myelination and cognition in young mice [322]

Abbreviations: CCH, chronic cerebral hypoperfusion; Chrm1, muscarinic acetylcholine receptor 1; CNPase, 2′,3′­cyclic nucleotide 3′­phosphodiesterase; HO‐1, heme oxygenase‐1; MAG, myelin‐associated glycoprotein; MBP, myelin basic protein; NG2, neuron‐glia antigen‐2; NRF2, nuclear factor erythroid 2‐related factor 2; Olig2, oligodendrocyte transcription factor 2; OPC, oligodendrocyte precursor cells; RXR, receptor retinoid X receptor; WMI, white mater injury.

#### 4.1.7 Physical Activity

It has been reported that PA may help delay the progression of dementia [323]. The underlying mechanisms may include the antibrain aging effect and anti‐AD effect, which has been described [324]. Recent studies have explored the role of PA in the recovery of myelin in AD. Constraint‐induced movement therapy has been shown to induce the maturation and differentiation of oligodendrocytes and promote myelin remodeling by inhibiting RhoA/ROCK2 signaling [325]. The activation of this pathway is associated with various risk factors for AD, including Aβ aggregation, tau phosphorylation, neuroinflammation, and synaptic damage, all of which contribute to the pathogenesis of AD [326]. Furthermore, voluntary running exercise has been reported to mitigate the loss of myelinated fibers and the demyelination of myelin sheaths in the dentate gyrus [327], CA1 [328], and medial PFC [329], thereby enhancing learning and memory abilities in APP/PS1 mice. Graham et al. [330] found the aerobic exercise ameliorated cognitive deterioration and appeared to keep components of myelin sheath and OPCs stabilized, resulting in a decrease in the percentage of loose and granulated myelin sheath and MBP protein, and an increase in NG2 mRNA and protein levels. Its underlying mechanism may include enhancing mitochondrial energy production and synaptic function [331], decreasing the inflammation caused by astrocyte secretions (TNF‐α, Cox‐2, CXCL2) associated with downregulation of miR‐34a and activation of TAN1/PI3K/CREB signaling pathway [148], and also reducing inflammation in the hippocampus and improves axons and myelin crosstalk [332]. These results showed that PA have an important role in remyelination of AD. However, as alternatives for people with limited activity, some physical stimuli, including PBMT, TMS, and HBOT as new technologies that may be suitable for remyelination and driving neural activity in AD patients.

#### 4.1.8 Transcranial Magnetic Stimulation

TMS is known as an established modality of neuromodulation [333]. Studies in vivo and in vitro have provided compelling evidence that TMS is an effective tool for inducing myelination in demyelinating diseases [333, 334]. Yang et al. [335] showed that deep rTMS effectively improved spatial working memory by dampening microglia activation and rectifying cytokines levels (IL‐1β, IL‐6, and IL‐10) in the region of demyelination in the cerebral cortex. rTMS also can increase the number of newborn oligodendrocytes in adult mouse cortex by enhancing their survival [336]. Recently, Da et al. [46] found that combined visual and auditory gamma‐sensory stimulation (1 h per day for 6 months) may modulate neuronal network function in AD in part by reducing white matter atrophy and myelin content loss, especially in the hippocampus and entorhinal cortex. Also, there is evidence that TMS, extra virgin olive oil and S‐allyl cysteine alone or in combination, reduce oxidative stress, increasing antioxidant defenses and also lowering the clinical score in demyelinating diseases [337], which need further exploration in AD‐related demyelination.

#### 4.1.9 Photobiomodulation Therapy

PBMT, previously referred to as low‐level laser (light) therapy, employs laser or light as a noninvasive therapeutic modality. The existing evidence suggests that various treatment paradigms, including transcranial and systemic PBM, as well as the recently proposed remote PBM, may hold promise for the treatment of AD. PBMT has been shown to reduce the formation and accumulation of Aβ protein [338, 339], and it has been reported to exert additional beneficial effects in AD [340]. PBM exerts a range of biological effects, such as enhancing mitochondrial function [341], mitigating neuroinflammation induced by activated glial cells [342, 343], improving glymphatic drainage [344], regulating the gut microbiome [345], and directing the differentiation of adult hippocampal NSCs toward neurons, among other effects [346]. However, Sipion et al. [347] found it unable to demonstrate any therapeutic effect of PBM for AD in a randomized and fully blinded long‐term in vivo study, there was no significant differences between the groups in behavioral tests. Similarly, histological analyses showed no differences in amyloid load, neuronal loss or microglial response underwent low or high power 810 nm light, three times a week for 5 months from the first to the sixth month of life, which corresponding to the prodromal phase of the pathology [348]. The above paradoxical conclusion indicated that when considering PBMT as a potential therapeutic solution for treatment of AD pathology [348], multiple parameters of PBMT should be considered including the light source and wavelength(s), output power, irradiance, irradiation time, fluence or total energy (dose), operation mode (continuous or pulsed) irradiation, approach and site, number of treatment sessions and, and so forth. However, there is currently no evidence indicating that PBMT specifically targets OPCs and myelin repair in AD, which need further explored.

#### 4.1.10 Hyperbaric Oxygen Therapy

HBOT—the medical administration of 100% oxygen at environmental pressure greater than one atmosphere absolute (ATA)—is used clinically for a wide range of medical conditions. One of main mechanisms of HBOT is elevation of the partial pressure of oxygen in the blood and tissues as compared to simple oxygen supplementation [349, 350]. This allows five to ten times more oxygen to enter the blood plasma and to reach tissues suffering from low oxygen supply (following, e.g., brain injury, stroke, or vascular dysfunction). Recently, more and more evidence showed that ischemia and hypoxia as a common trigger for demyelination in AD [351, 352], and HBOT could improve cognitive functions and ameliorate the reduced brain glucose metabolism in AD patients [353]. It has summarized that the role of oxygen metabolism abnormality in AD and HBOT was a potential strategy for treating AD by alleviating various pathological aspects, including inhibition of apoptosis, improvement of mitochondrial function, stem cell proliferation, enhancement of antioxidant defense activity, reduction in neuroinflammation, and neuroprotection [95]. Additionally, we have well‐explained that ischemia and hypoxia was an important risk factors for demyelination and that loss of myelin integrity could be an upstream risk factor for neuronal Aβ deposition, the central neuropathological hallmark of AD [4]. Therefore, improving oligodendrocyte health and myelin integrity by HBOT could be a promising target to delay development and slow progression of AD. Further studies with more rigorous design will help to fully evaluate the clinical value of hyperbaric oxygen on cognition function in people with AD, especially in the early stage. Furthermore, when given HBOT, we should consider the oxygen concentration, duration of oxygen supply and oxygen pressure, and we should regularly evaluate the treatment effect so as to help ensure the safety and effectiveness of HBOT.

As mentioned above, the noninvasive therapies may be able to promote myelin regeneration. Among them, PA and TMS have shown potent cognitive improvement and myelin protection effects in AD models (Table 3). Current studies have shown that HBOT mainly corrects upstream pathology by targeting AD‐related hypoxia, and PBMT promotes myelin regeneration through diverse mechanisms. As a supplement to pharmaceutic therapies, noninvasive therapies are suitable for personalized medicine, but further optimization of parameters such as TMS frequency and HBOT oxygen pressure is needed for clinical translation, and strict clinical trials are required to verify long‐term efficacy and safety.

**TABLE 3:** Noninvasive physical or brain stimulation therapies for remyelination in AD and other cognitive impairment diseases.

Noninvasive therapy	Pathway and mechanism	Key effects	Experimental model
Voluntary running exercise	The volume of the myelin in the CA1 region↑	Spatial learning and memory ability↑	APP/PS1 mice [328]
Voluntary running exercise	MBP^+^ area↑, CC1^+^ cells↑, MBP^+^ area surrounding Aβ↑, cell density, branching complexity of PDGFα^+^ cells around Aβ↑ → OPCs maturation and differentiation in the mPFC↑	Spatial learning and cognitive memory↑	APP/PS1 mice [329]
Voluntary running exercise	Myelin surface↑, number of myelin‐microglial colocalization surfaces↑	Spatial working memory↑	Mice with obesity‐induced cognitive decline and white matter damage [330]
Treadmill exercise	MBP↑, Olig2↑, PDGFR‐α↑ → myelin regeneration in the cortex↑	Spatial Cognitive Performance↑	D‐gal aging model [148]
TMS	4 weeks deep rTMS → MBP↑, GST‐π↑ → mature oligodendrocyte↑	Spatial Working Memory↑	CPZ model [335]
Noninvasive, combined visual and auditory gamma‐sensory stimulation	Combined visual and auditory 40 Hz gamma‐sensory stimulation → total cerebral white matter volume↑, T1w/T2w ratio↓ in the entorhinal region	White matter atrophy↓ and myelin content↑	AD patients [46]

Abbreviations: AD, Alzheimer's disease; CPZ, cuprizone; GST‐π, pi form of glutathione‐S‐transferase; MBP, myelin basic protein; mPFC, medial prefrontal cortex; Olig2, oligodendrocyte transcription factor 2; OPC, oligodendrocyte progenitor cells; TMS, transcranial magnetic stimulation.

### 4.2 Therapeutic Strategies Targeting Myelin Injury in Other Central Nervous System Myelin‐Related Diseases

#### 4.2.1 Multiple Sclerosis

Disease‐modifying therapies such as immunomodulators and immunosuppressants, primarily reduce inflammatory responses by regulating the immune system, thereby controlling the recurrence and progression of MS. Currently, FDA‐approved immunomodulatory drugs such as IFN‐β, glatiramer acetate (GA), and mitoxantrone are available. Among these, IFN‐β and GA, serving as first‐line immunomodulatory agents for MS, regulate the disease process through distinct mechanisms [354]. IFN‐β primarily alleviates CNS inflammation by modulating T‐cell function [355]. The mechanism of GA is more complex. It not only induces immune tolerance and also it regulates T‐cell function to partially suppress inflammation at the lesion site [356]. Additionally, GA can directly act on antigen‐presenting cells and B cells, enhancing the secretion of neurotrophic factors, thereby exerting neuroprotective effects [356]. Mitoxantrone was evaluated in a double‐blind Phase II study of mitoxantrone in patients with SPMS with and without relapses (MIMS) [357]. However, risk of serious side effects such as cardiomyopathy and treatment‐related acute leukemia has substantially limited indication in clinical practice [358]. Likewise, immunosuppressants primarily target the core autoimmune pathology by suppressing abnormal immune responses. Immunosuppressive drugs (e.g., methotrexate, cyclophosphamide, and azathioprine) have been tested in progressive MS. Although on occasion some therapeutic effects (in subgroups or as trends) have been observed, convincing effects on disability progression have not been shown [359, 360].

Monoclonal antibodies (mAbs) in MS have precise targeting role such as anti‐CD20 mAbs (e.g., Rituximab, Ocrelizumab). Rituximab rapidly suppresses new lesion formation in relapsing‐remitting MS (RRMS) [361], and Ocrelizumab stabilizes disability progression and reduces serum neurofilament light chain levels [362]. In a noninferiority comparative effectiveness observational cohort study, results did not show noninferiority of treatment with rituximab compared with ocrelizumab. And rituximab was associated with a higher risk of relapses than ocrelizumab as administered in everyday practice. And the efficacy of rituximab and ocrelizumab administered at uniform doses and intervals is being further evaluated in randomized noninferiority clinical trials [363]. Also, Bruton's tyrosine kinase inhibitors (BTKis) targeting B‐cell and myeloid cell activation is one of the most promising drugs [364]. Several BTKi candidates are currently in Phase II and III trials, including Evobrutinib [364], Tolebrutinib [365], Fenebrutinib [366], and so forth, and continuous monitoring of trial outcomes will determine their future clinical application.

Therapies targeting oligodendrocytes promote myelin regeneration and repair. Current research has identified various substances and targets that can enhance OPC differentiation and myelin regeneration. LINGO1, a CNS‐specific membrane glycoprotein that suppresses oligodendrocyte differentiation and myelination and blockade of this protein may therefore be effective in promoting remyelination in a clinical setting [367]. Clinical trials, particularly those involving the anti‐LINGO‐1 mAb BIIB033, have demonstrated encouraging results in enhancing remyelination and improving clinical outcomes [368]. G protein‐coupled receptor 17 (GPR17) is a potential drug targets for promoting OPC differentiation. GPR17 antagonists by blocking GPR17 signaling, relieve this inhibition of the differentiation pathway, thereby promoting OPC differentiation and remyelination [369].

#### 4.2.2 Leukodystrophies

Current therapeutic strategies aim to slow disease progression, alleviate symptoms, and, where feasible, address the underlying genetic or metabolic defects. These approaches include dietary modulation, substrate reduction, cell replacement, and advanced genetic interventions.

Curcumin, a polyphenol derived from turmeric, has demonstrated potential to modulate pathological ER stress [370]. It alleviates ER retention and apoptosis induced by truncated myelin protein zero mutants [371] and corrects CFTR misfolding in models of cystic fibrosis [372]. In PLP1 transgenic mice, curcumin treatment increases oligodendrocyte density, enhances myelination, and improves motor function, in part by attenuating neuroinflammation [373]. In X‐ALD, Lorenzo's oil competitively inhibits synthesis of VLCFAs; it may reduce the risk of cerebral disease in asymptomatic boys but is not effective once cerebral disease is established [374, 375].

Allogeneic hematopoietic stem cell transplantation (HSCT) remains the standard of care for early‐stage cerebral X‐ALD, KD, and MLD [376, 377]. Therapeutic efficacy relies on donor‐derived myeloid cells migrating into the CNS to replace dysfunctional microglia, thereby clearing toxic metabolites and modulating the inflammatory milieu [378]. However, success is strictly time‐dependent—outcomes are optimal only prior to irreversible neurological damage—and matched sibling donors are preferred due to lower graft failure rates [379].

Gene therapy represents a promising curative approach by correcting the genetic defect at its source. Hematopoietic stem cells are harvested, transduced ex vivo with lentiviral vectors encoding the functional gene, and reinfused. This autologous approach substantially reduces the risk of graft‐versus‐host disease by using the patient's own cells. For X‐ALD, lentiviral delivery of wild‐type ABCD1 has demonstrated favorable safety and efficacy signals in clinical trials [380, 381]. Recombinant adeno‐associated viral (AAV) vectors enable direct gene delivery to CNS tissues; for example, SBT101 (AAV9‐hABCD1) restores ABCD1 expression and corrects biochemical abnormalities in Abcd1‐null mice and received FDA Fast Track designation in 2022 [382]. CRISPR/Cas9 and base editing offer potential for precision genome correction. Systemic delivery of homology‐independent targeted integration (HITI) systems or engineered base editors (e.g., NG‐ABE8e) has achieved efficient correction of pathogenic variants and reduction of VLCFAs in humanized X‐ALD mouse models [383, 384]. For PMD caused by PLP1 duplication, attenuation of PLP1 overexpression is a critical therapeutic goal; RNA interference (RNAi) and antisense oligonucleotides (ASOs) have shown efficacy in preclinical models [385].

#### 4.2.3 Vascular Dementia/Cerebral Small Vessel Disease

Currently, no approved therapies specifically target myelin regeneration in cSVD. Clinical management primarily emphasizes the prevention and control of vascular risk factors, whereas myelin‐protective strategies remain an active area of research.

Effective hypertension management represents the most important intervention for slowing the progression of white matter lesions in cSVD [386]. In contrast to large‐vessel stroke, thrombosis is not considered a primary pathogenic driver of cSVD. In addition, large‐scale randomized controlled trials specifically evaluating the effects of smoking cessation, diabetes management, statin therapy, and lifestyle interventions in patients with lacunar stroke remain lacking. Future therapeutic strategies aim to disrupt the self‐perpetuating cycle of chronic hypoxia and neuroinflammation. Therapeutic strategies that modulate microglial polarization and promote OPC differentiation, many of which were originally developed for MS, are being repurposed for cSVD with the goal of restoring white matter integrity.

#### 4.2.4 Psychiatric Disorders

Therapeutic approaches for psychiatric disorders are evolving from neurotransmitter modulation toward disease‐modifying strategies that aim to restore neural circuit integrity by promoting myelination. Notably, several existing psychotropic agents demonstrate promyelinating effects.

The atypical antipsychotic quetiapine enhances OPC maturation and attenuates myelin loss, effects that are partly mediated by the restoration of epigenetic H3K9me3 levels [251, 387]. The antidepressant fluoxetine facilitates OPC differentiation through modulation of the Wnt signaling pathway [388], whereas the mood stabilizer lithium upregulates myelin‐related gene expression by inhibiting GSK3β [389]. The antihistamine clemastine, acting as a muscarinic receptor antagonist, also promotes remyelination and has also been shown to reverse social avoidance behaviors in animal models of social isolation [390]. Furthermore, the antioxidant N‐acetylcysteine (NAC) protects OPCs from oxidative stress and demonstrates adjunctive therapeutic benefits in disorders such as SCZ and ASD [391].

Emerging therapeutic interventions targeting the gut–brain axis include probiotic supplementation (e.g., *Lactobacillus* strains) and fecal microbiota transplantation (FMT), which may confer therapeutic effects by modulating SCFA levels and attenuating neuroinflammation [392, 393]. Nonpharmacological modalities such as physical exercise and PBMT (typically delivered via near‐infrared light) enhance mitochondrial function and elevate brain‐derived neurotrophic factor (BDNF) expression, thereby providing additional neuroprotective and promyelinating benefits [394, 395].

#### 4.2.5 Traumatic Brain Injury

Therapeutic strategies for TBI primarily target two objectives: preventing secondary demyelination and overcoming the inhibitory postinjury microenvironment to promote endogenous repair. Promising investigational approaches encompass multiple modalities, including small‐molecule drugs, mAbs, cell transplantation, and surgical and rehabilitative interventions.

Pharmacological modulation of specific receptors and intracellular pathways represents a central therapeutic strategy. Antagonism of receptors, including the muscarinic M1 receptor (e.g., via clemastine) or the histamine H3 receptor, as well as modulation of GPR17, has been shown to stimulate OPC differentiation [272, 396]. Inhibition of the RhoA/ROCK pathway facilitates OPC migration, whereas suppression of Notch signaling promotes OPC maturation, thereby contributing to enhanced remyelination [397]. Furthermore, the small‐molecule epigenetic‐silencing inhibitor ESI1 promotes myelin formation and has demonstrated efficacy even in aged animal models [398].

Neutralizing antibodies offer high‐specificity targeting capabilities against key inhibitory factors. Antibodies against Nogo‐A, a potent myelin‐associated inhibitor of regeneration, promote axonal sprouting and neural plasticity [399]. Antibodies targeting CD11d, HMGB1, or IL‐20 reduce pathological leukocyte infiltration and mitigate glial scar formation [272, 400]. The recombinant IgM antibody rHIgM22 binds CNS myelin, stimulates microglial phagocytosis of myelin debris, and thereby facilitates the clearance of inhibitory barriers to OPC‐mediated repair [401].

Transplantation of myelin‐competent cells aims to directly replace oligodendrocytes lost after injury [402, 403]. Several cellular sources are currently under investigation. Although derived from the peripheral nervous system, transplanted Schwann cells can promote axonal regeneration and remyelination within the CNS. Autologous Schwann cell transplantation has demonstrated feasibility and safety in a Phase I trial for subacute SCI [404, 405]. Olfactory ensheathing cells share biological properties with both Schwann cells and astrocytes and support axonal regrowth and myelination [406]. NSCs/neural stem/progenitor cells are multipotent and can differentiate into oligodendrocytes, neurons, and astrocytes, providing multimodal reparative support [407, 408]. Although direct OPC transplantation exhibits strong remyelination potential [409], it faces substantial challenges, including difficulties in obtaining high‐purity populations [410], potential glioma transformation risk [411], and suppression by the inhibitory injury microenvironment [412].

Surgical treatment and rehabilitation are receiving growing attention. Early decompression (< 24 h) is critical for attenuating secondary injury and improving neurological outcomes [413]. Emerging approaches in neuroregenerative surgery integrate hydrogel scaffolds directly into lesion sites to support neural regrowth [414]. Physical exercise represents a potent physiological stimulus for remyelination [415]. Advanced neuroengineering approaches, including brain–computer interfaces and virtual reality, are reshaping rehabilitation by actively promoting neuroplasticity [416].

## 5 Conclusions and Future Perspectives

Myelin dysfunction and related disorders in the CNS are highly complex and involve multiple diseases and pathological mechanisms. Genetic factors and environmental factors contribute to myelin impairment in AD and other CNS myelin‐related disorders. Myelin impairment in AD, MS, leukodystrophies, vascular dementia and cSVD, psychiatric disorders, and traumatic CNS injuries has distinct characteristics but also shares commonalities in terms of pathological mechanisms related to oligodendrocyte injury, myelination or maintenance impairment.

This convergence reveals a compelling therapeutic opportunity that transcends traditional disease classifications. Whether the primary insult arises from aging in AD, autoimmune attack in MS, specific genetic defects in leukodystrophies, chronic hypoperfusion in cSVD, neurodevelopmental and inflammatory perturbations in psychiatric conditions, or secondary injury cascades following CNS trauma, the downstream pathology often involves oligodendrocyte death, impaired differentiation of OPC, and failed remyelination. Accordingly, strategies that protect and regenerate myelin may have broad translational applicability. In AD, we summarized both pharmaceutic and noninvasive therapies. Pharmaceutic treatments act through various mechanisms to promote myelin regeneration, such as enhancing OPC differentiation, maturation of oligodendrocytes, and myelin formation. Noninvasive therapies like PA, TMS, PBMT, and HBOT also show potential in promoting myelin regeneration and improving cognitive function. And current research in other diverse disease models is focused primarily on two complementary approaches: (1) pharmacological interventions—including receptor modulators, signaling pathway‐targeted agents, and epigenetic regulators, as well as mAbs; and (2) cell transplantation strategies designed to directly supplement or replace damaged cells, such as OPC transplantation, NSC therapies, and HSCT. Collectively, these strategies aim to enhance myelin plasticity and facilitate functional recovery.

In conclusion, myelin dysfunction plays an important role in a range of CNS demyelinating diseases. Advancing our understanding of myelin's involvement has the potential to transform our comprehension of the disease's etiology and progression, while also driving the development of novel diagnostic methodologies and therapeutic approaches. Especially in AD, myelin impairment is an early pathological event. Focusing on myelin protection and regeneration, particularly when integrated with early diagnosis and precision interventions, is poised to become a fundamental component of future comprehensive AD treatment paradigms.

## Author Contributions

**Weidong Le and Lihong Huang** conceived the review. **Lihong Huang** searched the papers mainly related to Alzheimer disease and drafted the manuscript. **Zhen Hong and Peng Cheng** added five new chapters mainly related to MS, leukodystrophies, vascular dementia and cerebral small vessel disease, myelin abnormalities in psychiatric disorders, secondary myelin loss following traumatic CNS injury. **Guangdong Liu and Yang Xiang** helped for the acquisition of data. **Weidong Le, Zhen Hong, and Yang Xiang** helped to edit and revise the manuscript. All the authors have read and approved the manuscript.

## Funding

This study was supported by grants from National Science and Technology Innovation 2030 Major program of “Brain Science and Brain‐Like Research” 2021, Grant/Award (ID: 2022ZD0211600) ; from Sichuan Province Cadre Healthcare Research Project, Grant/Award (ID: 2025‐203); from Project of Department of Science and Technology of Sichuan Province, Grant/Award (ID: 2023YFS0267).

## Ethics Statement

The authors have nothing to report.

## Conflicts of Interest

The authors declare no conflicts of interest.

## Data Availability

The authors have nothing to report.
